# Supplementary material for: Neural mechanisms of economic commitment in the human medial prefrontal cortex
Source: eLife. 2014 Oct 21;3:e03701. doi: 10.7554/eLife.03701 (PMC4227044; doi:10.7554/eLife.03701)
Supplement: Figure 4—source data 1. — Columns show cluster and peak statistics as well as the x, y, z coordinates of the peaks. DOI: http://dx.doi.org/10.7554/eLife.03701.008 [file elife03701s001.docx]

**Figure 4 – source data**

| Cluster | Cluster | Cluster | Cluster | Peak | Peak | Peak | Peak | Peak |  |  |  |
| --- | --- | --- | --- | --- | --- | --- | --- | --- | --- | --- | --- |
| p (FWE) | p (FDR) | equivk | p (unc) | p (FWE) | p (FDR) | T | equivZ | p (unc) | x | y | z |
| 0.0000 | 0.0000 | 94 | 0.0000 | 0.0000 | 0.018 | 11.67 | 6.25 | 0.000 | -38 | 16 | 2 |
|  |  |  |  | 0.0000 | 0.018 | 11.23 | 6.15 | 0.000 | -34 | 20 | -10 |
| 0.0000 | 0.0000 | 145 | 0.0000 | 0.0000 | 0.018 | 11.34 | 6.17 | 0.000 | 30 | 24 | -6 |
|  |  |  |  | 0.0000 | 0.018 | 10.73 | 6.03 | 0.000 | 46 | 16 | 2 |
|  |  |  |  | 0.0010 | 0.148 | 8.38 | 5.36 | 0.000 | 54 | 12 | 6 |
| 0.0000 | 0.0000 | 185 | 0.0000 | 0.0000 | 0.018 | 11.19 | 6.14 | 0.000 | -10 | 4 | -2 |
|  |  |  |  | 0.0000 | 0.018 | 10.94 | 6.08 | 0.000 | 10 | -16 | 10 |
|  |  |  |  | 0.0000 | 0.018 | 10.83 | 6.05 | 0.000 | -10 | -16 | 10 |
| 0.0000 | 0.0000 | 318 | 0.0000 | 0.0000 | 0.018 | 10.86 | 6.06 | 0.000 | 18 | -76 | 50 |
|  |  |  |  | 0.0000 | 0.061 | 9.31 | 5.65 | 0.000 | 38 | -84 | 18 |
|  |  |  |  | 0.0000 | 0.084 | 8.99 | 5.55 | 0.000 | 50 | -52 | -18 |
| 0.0000 | 0.0000 | 147 | 0.0000 | 0.0000 | 0.032 | 10.14 | 5.88 | 0.000 | -50 | -36 | 42 |
|  |  |  |  | 0.0010 | 0.15 | 8.34 | 5.35 | 0.000 | -34 | -44 | 34 |
|  |  |  |  | 0.0020 | 0.154 | 8.18 | 5.29 | 0.000 | -30 | -56 | 54 |
| 0.0000 | 0.0000 | 254 | 0.0000 | 0.0000 | 0.032 | 10.1 | 5.87 | 0.000 | 6 | 28 | 30 |
|  |  |  |  | 0.0000 | 0.036 | 9.93 | 5.82 | 0.000 | -2 | 32 | 38 |
|  |  |  |  | 0.0000 | 0.046 | 9.63 | 5.74 | 0.000 | 2 | 24 | 42 |
| 0.0000 | 0.0000 | 120 | 0.0000 | 0.0000 | 0.036 | 9.86 | 5.8 | 0.000 | -42 | -72 | -10 |
|  |  |  |  | 0.0010 | 0.138 | 8.49 | 5.4 | 0.000 | -42 | -84 | -10 |
|  |  |  |  | 0.0010 | 0.15 | 8.32 | 5.34 | 0.000 | -34 | -88 | 10 |
| 0.0000 | 0.0000 | 30 | 0.0000 | 0.0000 | 0.061 | 9.36 | 5.66 | 0.000 | -42 | 52 | 6 |
| 0.0000 | 0.0000 | 29 | 0.0000 | 0.0010 | 0.138 | 8.5 | 5.4 | 0.000 | -2 | -80 | -2 |
|  |  |  |  | 0.0040 | 0.204 | 7.8 | 5.16 | 0.000 | -10 | -76 | 10 |
| 0.0000 | 0.0000 | 21 | 0.0000 | 0.0020 | 0.155 | 8.11 | 5.27 | 0.000 | 22 | 16 | 62 |
|  |  |  |  | 0.0080 | 0.318 | 7.4 | 5.02 | 0.000 | 26 | 8 | 62 |
| 0.0000 | 0.0000 | 10 | 0.0000 | 0.0040 | 0.204 | 7.79 | 5.16 | 0.000 | -2 | -24 | 26 |
| 0.0000 | 0.0010 | 8 | 0.0010 | 0.0120 | 0.431 | 7.19 | 4.94 | 0.000 | 18 | -96 | -2 |
| 0.0010 | 0.0150 | 4 | 0.0100 | 0.0140 | 0.468 | 7.12 | 4.91 | 0.000 | 14 | -72 | 10 |
| 0.0010 | 0.0150 | 4 | 0.0100 | 0.0220 | 0.637 | 6.9 | 4.83 | 0.000 | 42 | 44 | 22 |
| 0.0020 | 0.0300 | 3 | 0.0230 | 0.0230 | 0.64 | 6.87 | 4.81 | 0.000 | 46 | 48 | 6 |
| 0.0150 | 0.1620 | 1 | 0.1620 | 0.0260 | 0.662 | 6.82 | 4.79 | 0.000 | -38 | -56 | -34 |
| 0.0150 | 0.1620 | 1 | 0.1620 | 0.0300 | 0.713 | 6.75 | 4.76 | 0.000 | 42 | 36 | 6 |
| 0.0150 | 0.1620 | 1 | 0.1620 | 0.0390 | 0.844 | 6.62 | 4.71 | 0.000 | -42 | 28 | 30 |
| 0.0150 | 0.1620 | 1 | 0.1620 | 0.0400 | 0.852 | 6.61 | 4.71 | 0.000 | 38 | -56 | -30 |
| 0.0150 | 0.1620 | 1 | 0.1620 | 0.0470 | 0.952 | 6.53 | 4.67 | 0.000 | 42 | 32 | 38 |
